# Lipoproteins and Cardiovascular Disease: An Update on the Clinical Significance of Atherogenic Small, Dense LDL and New Therapeutical Options

**DOI:** 10.3390/biomedicines9111579

**Published:** 2021-10-29

**Authors:** Ali A. Rizvi, Anca Pantea Stoian, Andrej Janez, Manfredi Rizzo

**Affiliations:** 1 Department of Medicine, University of Central Florida College of Medicine, Orlando, FL 32827, USA; ali.rizvi@ucf.edu; 2 Division of Endocrinology, Diabetes and Metabolism University of South Carolina School of Medicine Columbia, Columbia, SC 29209, USA; 3 Department of Diabetes, Nutrition and Metabolic Diseases, Carol Davila University of Medicine, 050474 Bucharest, Romania; ancastoian@yahoo.com; 4 Department of Endocrinology, Diabetes and Metabolic Diseases, University Medical Centre Ljubljana, Faculty of Medicine, University of Ljubljana, 1000 Ljubljana, Slovenia; andrej.janez@kclj.si; 5 Department of Health Promotion, Mother and Child Care, Internal Medicine and Medical Specialties (Promise), University of Palermo, 90133 Palermo, Italy

**Keywords:** small, dense LDL, prevention, cardiovascular risk, therapy, diabetes, GLP1

## Abstract

Dyslipidemia is a potent risk factor for the genesis and progression of cardiovascular disease (CVD), and both the concentration and type of low-density lipoproteins (LDL) augment this association. The small, dense LDL (sdLDL) subfraction is the subtype which is most strongly predictive of atherosclerosis and cardiovascular events. In addition to the traditionally available lipid-lowering treatment options, certain novel therapies have been shown to favorably impact sdLDL, among them the antidiabetic class of agents known as glucagon-like peptide 1 receptor agonists (GLP1-RAs). These drugs seem to alter the pathophysiologic mechanisms responsible for the formation and accumulation of atherogenic lipoprotein particles, thus potentially reducing cardiovascular outcomes. They represent a uniquely targeted therapeutic approach to reduce cardiometabolic risk and warrant further study for their beneficial nonglycemic actions.

## 1. Introduction

Cardiovascular disease (CVD) is the leading cause of death globally, causing an estimated 18 million deaths each year [1], and hyperlipidemia is a major risk factor for the development and progression of atherosclerotic CVD [1]. Low-density lipoprotein (LDL)-cholesterol has been identified as a key target for management and prevention of CVD [2]. Clinical interest has focused recently on a common human atherogenic lipoprotein phenotype characterized by a moderate increase in plasma triglycerides, a decrease in high density lipoprotein (HDL) cholesterol and the prevalence of small dense LDL (sdLDL) subspecies [3].

Despite adherence to current standard treatment guidelines, many patients with atherogenic dyslipidemia display a high residual risk of atherosclerotic cardiovascular events [4]. The atherogenic process is also due to the increased number of apolipoprotein B (apoB)-containing particles characterized by elevated non-HDL-C levels. LDL cholesterol-to-apo B ratio is a potential indicator of LDL particle size and plasma atherogenicity in type 2 diabetes (T2DM) as well as a potential indicator of increased cardiovascular risk in these patients. [5]. The emerging role of the retinol-binding protein 4 (RBP4) in the metabolism of sdLDL is also being increasingly recognized, since changes in RBP4 levels are associated with those in the apolipoprotein B-containing lipoproteins during dietary and drug treatment [6,7]. The current brief report is designed to provide an update on recent knowledge gained in this field.

## 2. Atherogenicity of Small, Dense LDL

Up to seven distinct LDL subspecies have been historically identified by nondenaturing gradient gel electrophoresis and ultracentrifugation based on density, size, charge, and the lipid and apoprotein content [8]. Ethnicity and genetics seem to be associated with LDL heterogeneity [9,10,11]. Smaller LDL particles have a greater propensity for uptake by arterial tissue than larger subspecies [8], suggesting greater transendothelial transport. Small, dense LDL also has decreased receptor mediated uptake, increased proteoglycan binding, and more susceptibility to oxidation [8,12]. The latter has been linked to a number of factors that are peculiar to sdLDL, such as altered properties of the surface lipid layer associated with reduced content of free cholesterol, diminished antioxidant content, and increased content of polyunsaturated fatty acids [12].

The predominance of sdLDL is associated with a significant increased risk of coronary artery disease. This association was demonstrated several years ago in case control studies of subjects with myocardial infarction [13,14,15], and confirmed in studies performed on patients with angiographically documented coronary disease [16]. Prospective studies of the relation of LDL size with the development of coronary artery disease have been carried out, including the first pioneer nested case-control analysis in the Physician′s Health Study [17], the population-based Stanford Five Cities Project [18], and the Quebec Cardiovascular Study [19]. These studies clearly showed that reduced LDL size was a significant univariate predictor of coronary disease. These findings were later confirmed in many subsequent studies, and critically reviewed by the European panel of experts’ discussions on LDL subclasses [20]. It seems the all the metabolic changes associated with the production of sdLDL collectively contribute to cardiometabolic risk, and increased number of atherogenic LDL particles must be present for disease risk to be evident.

## 3. The Clinical Significance of Small, Dense LDL

**Figure 1:**
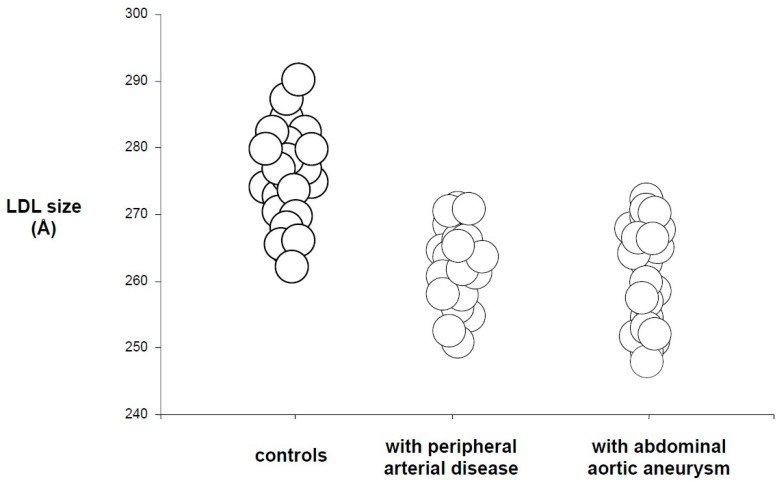
LDL size in patients with peripheral arterial disease, abdominal aortic aneurysm and controls (adapted from [23,24]).

All the above-mentioned characteristics make sdLDL particles highly atherogenic and closely linked to the early stages of subclinical atherosclerosis and endothelial dysfunction, thus enhancing the risk of cardiovascular events [21]. Predominance of sdLDL particles is associated with increased risk of coronary artery disease, as demonstrated in case control studies, large epidemiological studies, clinical intervention trials, and angiographic studies [22]. Therefore, sdLDL is recognized as an emerging risk factor for CVD [23] since it is a major lipid alteration seen in patients with coronary artery disease, peripheral arterial disease, abdominal aortic aneurysm, diabetes, the metabolic syndrome and other categories of patients at high CV risk [24,25,26,27,28] (Figure 1).

The evidence for sdLDL as a crucial factor for the development and progression of atherosclerosis stems from the observation that the initial discernible accumulation of lipids in the arterial (the so-called “fatty streak”) consists of the accumulation of foam cells which have a high sdLDL content [29]. Because of reduced affinity to LDL receptor, sdLDL tends to circulate for a longer period in the blood stream. In addition, due to their distinctive physico-chemical composition, these LDL particles have greater arterial uptake and retention. They are able to penetrate into the vascular intima and are quickly transformed into oxidized LDL because of high susceptibility to oxidation [8,20]. An increased carotid artery intima-media thickness (IMT) is considered one of the best surrogate markers of early “subclinical” atherosclerosis, and has been shown to correlate significantly with the presence of coronary heart disease and to predict coronary events [30]. Studies have demonstrated sdLDL is independently associated with carotid IMT [31].

## 4. Small, Dense LDL, Insulin Resistance and Diabetes

We are in the midst of an inexorable increase in the incidence of T2DM worldwide [32]. The atherogenic lipoprotein phenotype manifested by hypertriglyceridemia, low HDL-cholesterol concentrations and elevated levels of sdLDL particles [3] is predominant lipid abnormality in subjects with insulin resistance, T2DM and the metabolic syndrome [33]. The genesis of sdLDL is closely linked to the presence of hypertriglyceridaemia, and Berneis and Kaspar have proposed two different pathways for the formation of sdLDL, according to hepatic triglyceride availability [8]. The secretion of very-low density lipoproteins (VLDL) by the liver is followed by the action of specific enzymes, first by lipoprotein lipase, with the production of remnant particles, intermediate density lipoproteins or larger LDL, and then by hepatic lipase, with the production of LDL subspecies with smaller size and higher density [8]. Another enzyme, the cholesteryl ester transfer protein (CETP), which usually collects triglycerides from VLDL or chylomicrons in exchange of cholesteryl esters from HDL, is able to transfer triglycerides to LDL, that are further processed by hepatic lipase into sdLDL [8]. Small, dense LDL are typically formed in states of hypertriglyceridaemia [8], and treatment of the latter, including that originating secondarily from suboptimally controlled diabetes, is often appropriate management of an individual with high levels of sdLDL [20]. An association of LDL particle size with the cluster of risk factors that characterize the insulin resistance syndrome has been demonstrated [34], and there is strong evidence that the smaller denser LDL particles can be added to the group of cardiometabolic alterations described as the metabolic syndrome [35].

It follows that the risk factor profile of subjects with a predominance of sdLDL is similar to that observed in states of insulin resistance, T2DM or the metabolic syndrome. It also seems that some adipokines, such as resistin, are closely associated with LDL heterogeneity [36]. A pioneer nested case control study of 204 elderly men and women from Finland clearly demonstrated that subjects with elevated levels of sdLDL had the greatest risk for developing T2DM over a 3.5-year follow-up period, independently of age, gender, glucose tolerance and body mass index [37]. Of interest, in this study a significant decrease in the risk of T2DM was associated with a very slight increase in LDL diameter [37], providing the basis for an effective cardiometabolic prevention by targeting atherogenic LDL particles. This link between atherogenic profile, insulin resistance and T2DM is explained by the effects of insulin and triglycerides on VLDL production and secretion, hepatic lipase activity and the resulting remodeling of triglyceride-enriched LDL particles to denser and more atherogenic LDL subspecies [20].

## 5. Managing Small, Dense LDL to Reduce Cardiometabolic Risk

The clinical significance of sdLDL has gained increasing interest for current-day management of cardiometabolic risk. and the question has been raised whether cardiometabolic treatments may favorably impact particle atherogenecity. Beyond the known effects of traditional lipid-lowering and oral glucose-lowering drugs [38,39], novel antidiabetic therapies with cardiovascular benefit may also reduce sdLDL concentrations [40]. The latter include three main categories of innovative drugs: (a) dipeptidyl peptidase 4 inhibitors (DPP-4i); (b) glucagon like peptide 1 receptor agonists (GLP-1RAs) and (c) sodium-glucose co-transporter-2 inhibitors (SGLT-2i). Cardiovascular safety has been shown with the use of DPP-4i, while GLP-1RAs and SGLT-2i have shown significant cardiovascular benefit. The last two classes of drugs are recommended as first-line antidiabetic therapies in high-risk individuals by current international guidelines [41].

Of interest, glucagon-like peptide 1 receptor agonists (GLP1-RAs) have shown favorable effects on lipids. They reduce lipoprotein and chylomicron production, as well as postprandial triglycerides, in patients with T2DM [42]. In addition, preclinical studies in mice models with T2DM have shown that certain GLP1-RAs, such as liraglutide, have lipid-lowering effects due to their action on hepatic liposynthesis, with a consequent reduction in triglycerides levels [43]. Both fasting and postprandial triglycerides levels seem to be modulated by liraglutide [44,45]. Of important clinical relevance is that metabolism of triglycerides in T2DM has a primary role in cardiovascular risk; indeed, longitudinal studies have shown that plasma triglycerides represent independent predictors of cardiovascular mortality in T2DM patients [46]. As discussed above, triglyceride-rich lipoproteins are the precursors of sdLDL and, therefore, of great clinical significance in patients with various cardiometabolic disorders [23]. Liraglutide has been shown to reduce such atherogenic lipoproteins [47,48].

In a randomized, double-blind, placebo-controlled, cross-over trial, the combination of liraglutide and metformin reduced both total LDL subfractions and sdLDL in patients with stable coronary artery disease and newly diagnosed T2DM [47]. These findings were confirmed in a real-world setting when the vascular benefit of liraglutide in patients with T2DM was associated with reductions in sdLDL, independent of glycemic control and body weight reduction [49]. This evidence points to a direct role of liraglutide in atherosclerosis formation and progression, supporting the postulated anti-atheroscletotic role of this agent [29]. Emerging clinical data appear to confirm the pioneering findings from preclinical studies that showed a reduction of intracoronary plaque and a decrease in myocardial infarct size with the use of liraglutide [49,50].

In summary, the direct action of GLP1-RAs on sdLDL, as exemplified by liraglutide, is most likely due to their modulation of the pathophysiological alterations responsible for the proatherogenic activity of lipoprotein subfractions [51]. Of note, it may represent one of the key mechanisms by which GLP1-RAs are able to reduce cardiovascular events and mortality [29,52].

## 6. Conclusions

It is becoming increasingly evident that sdLDL is both a strong predictor of coronary artery disease severity as well as a treatment target for the prevention of future CV events. It appears to confer an additional amount of risk stratification beyond that obtained from the well-recognized lipid-based parameters such as total and LDL cholesterol values. In addition, elevated sdLDL levels seem to be a consistent lipid abnormality in subjects with insulin resistance, T2DM and the metabolic syndrome. These data reinforce the importance of both the quantity and the quality of LDL for effective management of cardiometabolic risk. Finally, the newer classes of antidiabetic agents such as GLP1-RAs exert favorable effects on sdLDL through mechanisms that are only partially understood at this point. By contrast, contradictory findings on the lowering effect of sdLDL have been shown with the use of CETP inhibitors. Evacetrapib, as monotherapy or with statins, significantly reduced the concentrations of atherogenic apoB-containing particles, including sdLDL [53], while opposite to that, anacetrapib produced significant reductions in total LDL particles and all LDL subfractions, except for increases in the smallest, most dense subspecies [54,55]. This field is ripe for further elucidation and progress in order to streamline future therapeutic options and translate them into clinical benefits.

## Data Availability

Not applicable.
